# Motor control pathways in the nervous system of *Octopus vulgaris* arm

**DOI:** 10.1007/s00359-019-01332-6

**Published:** 2019-03-27

**Authors:** Letizia Zullo, Hadas Eichenstein, Federica Maiole, Binyamin Hochner

**Affiliations:** 1 Center for Synaptic Neuroscience and Technology, Istituto Italiano di Tecnologia, Largo Rosanna Benzi, 10, Torre D1, 16132 Genoa, Italy; 2 Department of Neurobiology, Silberman Institute of Life Sciences, Hebrew University, Jerusalem, Israel; 3 University of Genova, Viale Benedetto XV, 3, 16132 Genova, Italy; 4 IRCCS Ospedale Policlinico San Martino, Genova, Italy

**Keywords:** Octopus, Arm, Motor control, Peripheral nervous system, *En passant*

## Abstract

The octopus’s arms have virtually infinite degrees of freedom, providing a unique opportunity for studying movement control in a redundant motor system. Here, we investigated the organization of the connections between the brain and arms through the cerebrobrachial tracts (CBT). To do this, we analyzed the neuronal activity associated with the contraction of a small muscle strand left connected at the middle of a long isolated CBT. Both electrical activity in the CBT and muscle contraction could be induced at low threshold values irrespective of stimulus direction and distance from the muscle strand. This suggests that axons associated with transmitting motor commands run along the CBT and innervate a large pool of motor neurons *en passant*. This type of innervation implies that central and peripheral motor commands involve the simultaneous recruitment of large groups of motor neurons along the arm as required, for example, in arm stiffening, and that the site of movement initiation along the arm may be determined through a unique interplay between global central commands and local sensory signals.

**Electronic supplementary material:**

The online version of this article (10.1007/s00359-019-01332-6) contains supplementary material, which is available to authorized users.

## Introduction

**Fig. 1:**
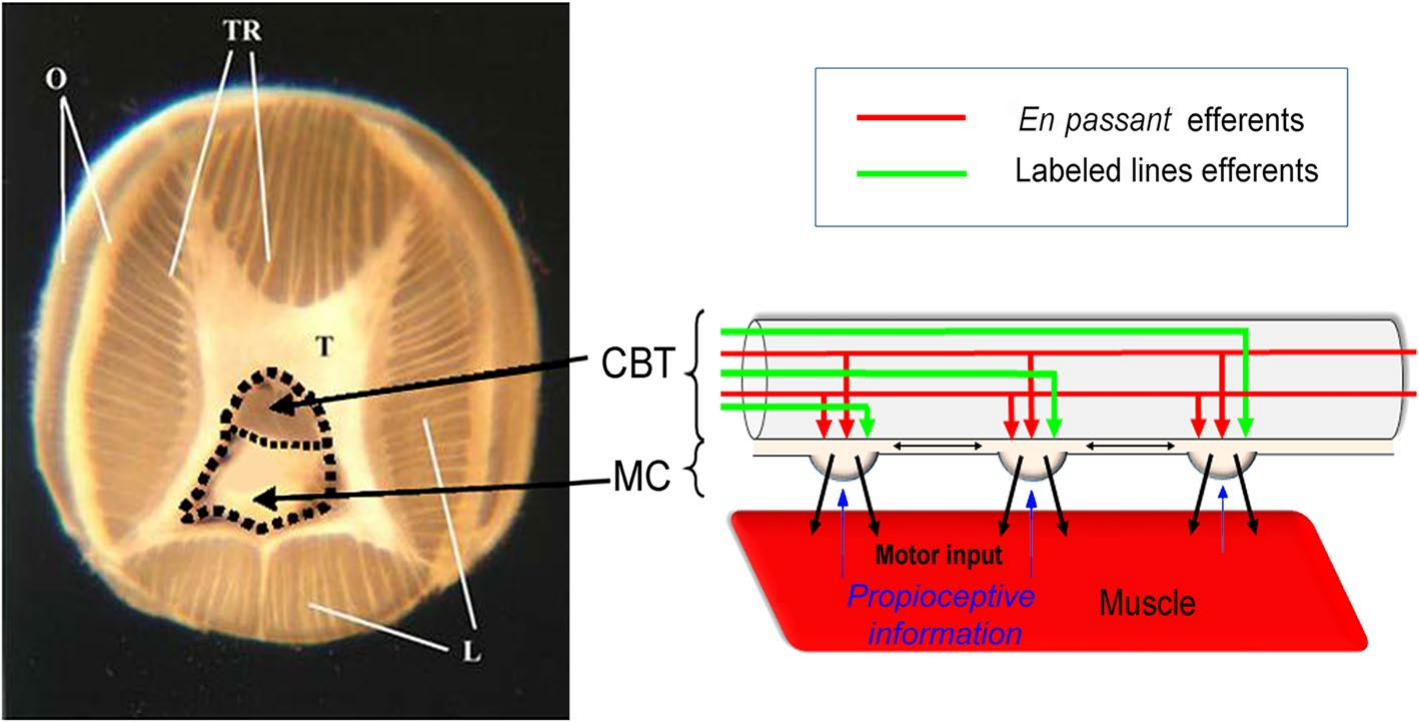
Possible models of the axial nerve cord (ANC) transmission pathway. Transverse section of an octopus arm where ANC (thick dashed lines) with cerebrobrachial tracts (CBT), medullary cord (MC) and the various type of muscles (transverse, T; longitudinal, L; TR, trabeculae, O, oblique) are shown. Possible models of functional configurations of *en passant* (red) and labeled lines (green) are schematized in lateral view (see text for explanation)

Octopus arms perform both motor and sensory functions essential for the interaction of the octopus with its external environment. The arms lack a rigid skeleton and are composed of a tightly packed array of small muscle fibers within the connective tissue matrix (Kier and Smith 1985; Kier and Stella 2007; Feinstein et al. 2011; Kier 2016). These muscles are arranged in the cylinder-like structure of the arm in three main groups: oblique (O), longitudinal (L) and transverse muscles (T) including also the trabeculae (TR) (Fig. 1). This structure is termed a “muscular hydrostat” due to the constant volume constraint that enables the antagonistic action of the different muscle groups (Kier and Smith 1985). This organization allows the arm musculature to produce both stiffened skeletal-like support and contraction force, the two components essential for movement generation (Levy et al. 2017). The muscular hydrostat structure dramatically increases the potential maneuverability of arms but imposes a huge load on the motor control system. Indeed, muscular hydrostats are redundant with a number of degrees of freedom far greater than those needed to define a single movement task, making movement control a problem of immense complexity. This complexity has encouraged research in the field of soft robotics in which soft-bodied animals serve as inspiration for the development of flexible continuum robotic arms (Guglielmino et al. 2012, 2013; Li et al. 2012; Kang et al. 2016; Nakajima et al. 2018). Investigation of the physiology of the octopus arm is also especially interesting from an evolutionary–developmental perspective and for comparative investigations in the field of regenerative biology (Fossati et al. 2013, 2015; Nodl et al. 2015; Sommese et al. 2017; Zullo et al. 2017, 2018a, b).

The arm nervous system consists of a prominent axial nerve cord (ANC), five intramuscular nerve cords and the ganglia of the suckers. The ANC is composed of two axonal tracts, the cerebrobrachial tracts (CBT), running dorsally along the ANC, and a medullary cord (MC) located beneath. The MC comprises a cellular cortex of unipolar nerve cell bodies surrounding an inner neuropil that expands in a local swelling opposite each sucker. The CBT contain axons that transmit efferent signals to the arm and afferent information to the CNS and local interganglionic connections (Graziadei 1971; Young 1971) [see scheme in Fig. 1; terminology is based on the suggestions of (Richter et al. 2010)]. The motor system axons from the brain do not directly innervate the arm muscles but send projections to the MC. The numerous roots emerging laterally from the MC swellings (ganglia) innervate a narrow area of intrinsic muscles of the arm that generates arm movement (Gutfreund et al. 2006).

Here, we investigate the basic organization of motor command transmission along the CBT. JZ Young has already pointed out that 180 million neurons in the central brain are connected with more than 40,000,000 neurons in each of the eight arms via relatively few efferents (~ 32,000) and afferents (~ 140,000). According to Graziadei and Young (Young 1971), about 380,000 motor neurons are distributed along the neuropil of the MC of each arm. A crude calculation suggests that roughly 1500 motor neurons innervate a 1-mm-long section of the arm (in an average arm of 250 mm length) (Levy et al. 2017).

This anatomical organization suggests that much of the sensory information and motor commands are processed in the peripheral nervous system of the arm (Fossati et al. 2011), while the brain sends inputs to activate specific motor actions. Indeed, stereotypical arm extensions can be elicited in denervated arms by stimulation of the arm CBT (Sumbre et al. 2001). In contrast to skeletal animals, the higher motor centers in the octopus central brain are not organized somatotopically and stimulation of these centers activates complex behaviors such as multi-arm extension (Zullo et al. 2009). These anatomical results and physiological findings prompt us to examine the functional organization of motor command transmission along the PNS. We tested whether the site of movement initiation along the arm is determined by axonal tracts organized in the CBT as “labeled lines” (see scheme in Fig. 1) allowing the brain to activate a peripheral program at a specific location along the arm. Surprisingly, our results do not support the involvement of labeled lines but rather suggest that axons running along the CBT innervate the motor neuron pools along the arm *en passant*.

## Materials and methods

### Animal treatments

Specimens of *Octopus vulgaris* were collected by local anglers from the Mediterranean Sea during winter–early summer. The octopuses were housed individually in 50 × 50 × 80 cm glass aquaria containing artificial seawater prepared with synthetic marine salt (Red Sea salt). The water was continuously circulated in a closed system and filtered through coral dust and active charcoal. Aquaria were regulated to 17 °C, a 12/12-h light/dark cycle, and the octopuses were fed with fish meat every second day. Animals were left to adapt to captivity for at least 10 days before use.

Artificial seawater was used as the experimental physiological solution: (in mM) NaCl, 460; KCl, 10; MgCl2, 55; CaCl2, 11; Hepes, 10; glucose, 10; pH 7.6. To obtain the isolated nerve cord preparation, animals were anesthetized in cold seawater supplemented with 1% ethanol and 55 mM MgCl2. A segment 7–10 cm long was cut from the middle of the arm and kept in oxygenated ice-cooled seawater for up to 20 min. Given the large portion of arm excised, after amputation the animals were not allowed to recover and were given terminal anesthesia with an overdose of ethanol, following requirements of the guidelines (Fiorito et al. 2015).

### Isolated axial nerve cord preparation

**Fig. 2:**
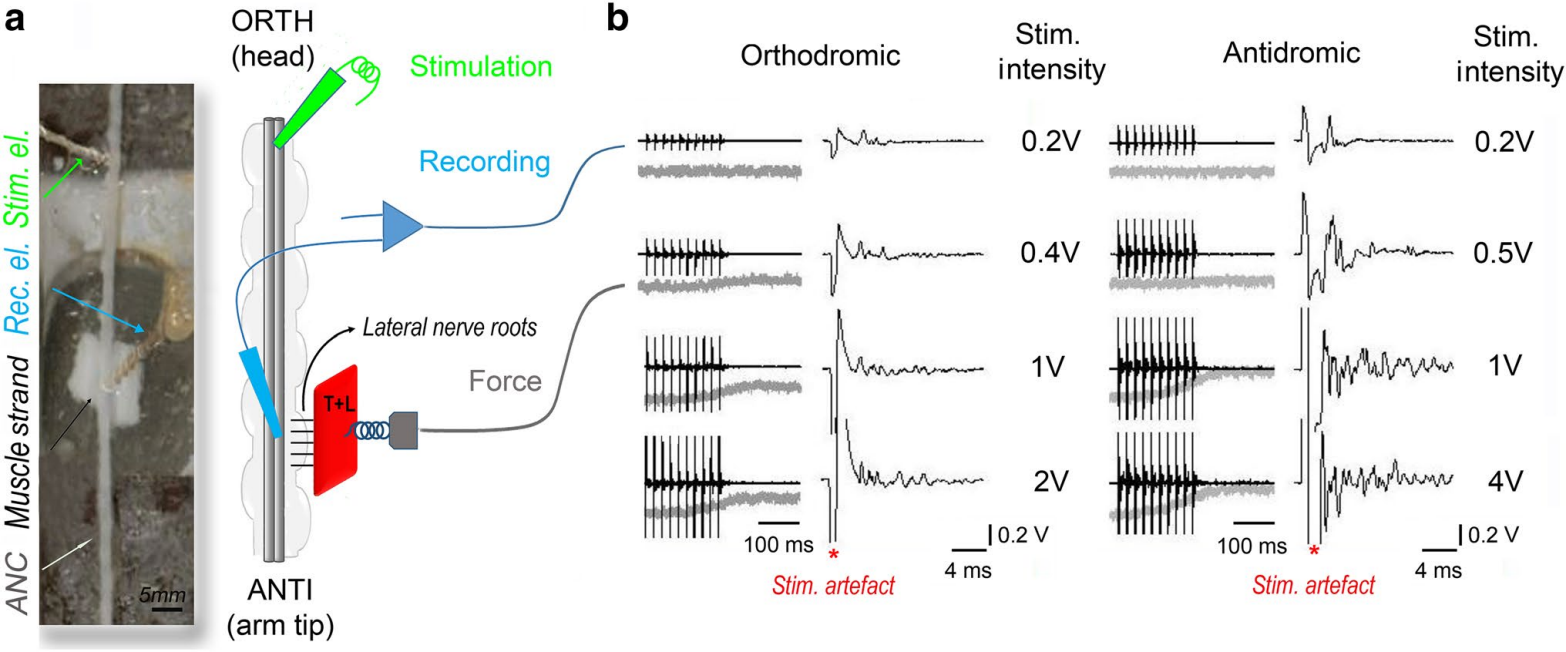
The experimental set-up. **a** Picture and schematic drawing of the preparation employed showing a long section of an isolated axial nerve cord (ANC) connected to muscle strands, containing both T and L muscles, hooked to a force transducer. The recording suction electrode (labeled in blue) was attached to one of the CBT at the level of the muscle strand. Trains of 10 pulses at 50 Hz of increasing strength were delivered from a suction electrode (green in **a**) positioned at different distances from the recording electrode at either orthodromic (ORTH) or antidromic (ANTI) orientation. **b** Examples of physiological results. Axonal tract (CBT) activity (black traces, shown at slow and fast time scale) and the force transducer output (gray traces) evoked by the stimulus intensity which is given for each recording trace. Force traces were uncalibrated and were used only to detect muscle contraction. The faster time sweeps of the CBT electrical activity in the second and fourth columns show the response to the last pulse in the train. Voltage scale bar refers to the electrical activity

The arm segments (*n* = 19) were dissected to expose and isolate the arm nerve cord. In each preparation (scheme in Fig. 2a), a strand of longitudinal and transverse muscles about 10 mm long and 2 mm wide was left connected at the middle of the isolated axial nerve cord (ANC). Care was taken to preserve the lateral nerves containing the motor neuron axons projecting from the medullary cord (MC) to the muscle strand. The muscle strand was innervated by about 50 nerve roots carrying the motor neuron axons to the intrinsic muscles (Graziadei 1971; Matzner et al. 2000; Gutfreund et al. 2006). The preparation was transferred to a Sylgard-coated recording chamber and continuously perfused with oxygenated ASW at ~ 18–20 °C (0.5 bath volume/min exchange).

Suction electrodes pulled from polyethylene tubing (opening of 50–150 µm) were used for extracellular recording and stimulation. A silver wire wrapped around the outside of the pipette served as a reference electrode for a silver wire recording/stimulation electrode inside the pipette. The recording electrode was placed at one of the two cerebrobrachial tracts (CBT) ipsilateral to the muscle strand examined. The recording electrode remained in place throughout the experiment while the stimulation electrode was repositioned along the same CBT to allow stimulating in its anterograde (orthodromic, ORTH) or retrograde (antidromic, ANTI) direction (Fig. 2). Activity evoked in the CBT was continuously recorded, amplified (×10.000) using a differential AC amplifier (Warner Instrument Corp. DP-304), bandwidth filtered at 300Hz–10 kHz, digitized, saved and analyzed with LabView 5 (National Instrument). Stimuli were 50 Hz trains of 10 pulses of 0.1 ms negative voltage step applied to the CBT. Stimulus intensity was set slightly above the response threshold and then gradually reduced to define the threshold intensity for evoking activity in the CBT and muscle contraction.

The muscle strand was connected with a stainless steel hook and silk thread to an isometric force transducer containing a strain-dependent resistor gauge (sensitivity 20 mV/g), custom made by the HUJI workshop, to monitor the tension generated across the muscle strand following electrical stimulation of the CBT. The transducer was pulled up by a micrometric manipulator until force imposed by the muscle strand was detected to ensure that muscle contraction was measured. The voltage output of the transducer showed muscle movement but we made no attempt to obtain quantitative force measurements. The transducer output was recorded, digitized and stored simultaneously with the CBT recordings. Data were clustered into two groups according to whether the stimulating electrode was positioned ORTH or ANTI to the recording site.

### Immunostaining

Arm samples were fixed in 4% PFA-ASW, embedded in OCT compound, serially sectioned at 40 µm on cryostat and collected onto Superfrost Ultra Plus (Menzel-Gläser). Sections were permeabilized in 1 × PBS + 1% Tween (PBS-T) two times for 5 min at room temperature (RT) and incubated in blocking solution (PBS-T + 10% normal goat serum) for 1 h at RT. The sections were labeled for neurofilaments (NF) by overnight incubation at 4 °C with mouse NF200 (SIGMA, diluted 1:100 in blocking solution). After three PBS-T washes for 10 min, sections were incubated in Alexa Fluor® 546 conjugated anti-mouse (1:1000 in blocking solution) for 2 h at RT. Tissues were rinsed several times and mounted in ProLong Gold antifade reagent (Life Technologies, Milan, Italy). Sections were imaged by inverted confocal laser microscope (SP8, Leica Microsystems GmbH, Wetzlar, Germany) and three-dimensional reconstructions were generated using Leica Application Suit X software (LAS-X).

### Statistics

The program SigmaPlot 13.0 (Systat Software, Inc.) was used for statistical analysis. Normality of the dataset was first assessed with normality test (Shapiro–Wilk). Parametric *t* tests and non-parametric Mann–Whitney rank sum test were used to compare datasets. In box plot analysis, for graphical reasons, outliers were not represented but they were included in the statistical analysis. Either linear or polynomial (log-normal three parameters) dynamic curve fitting was employed to visualize and plot the curve best describing the data. Dynamic curve fitting consists of an iterative process converging to the best possible solution. The tendency of the variables to increase or decrease together was tested with Pearson correlation giving the correlation coefficient (ρ) and *p* value. *p* < 0.05 was considered significant.

## Results

### Isolated axial nerve cord stimulation and recording

Figure 2 shows a scheme of the experimental set-up (a) with typical contraction outputs (gray traces in b) and recordings of electrical activity in the CBT (black traces in b) induced by orthodromic (ORTH) and antidromic (ANTI) electrical stimulation. Stimulation of the CBT evoked complex bursts of activity and contraction of the muscle strand located at both ORTH and ANTI to the stimulating electrodes (Fig. 2a). The threshold for evoking contraction was higher than that for evoking CBT activity (contraction threshold was 0.4V ORTH and 0.5V ANTI vs. 0.2V threshold for CBT activity in both configurations). This indicates that contraction requires the recruiting of more axons than needed to generate a recordable burst in the CBT. The fact that contraction can be evoked at lower stimulus intensities ortho- than antidromically may be due to differences in the effectiveness of stimulation (but note that the recording electrode was not moved throughout the experiment). Further interpretation of our data is, therefore, based on the statistical analysis of a large number of similar experiments and considering variability due to stimulation effectiveness.

**Fig. 3:**
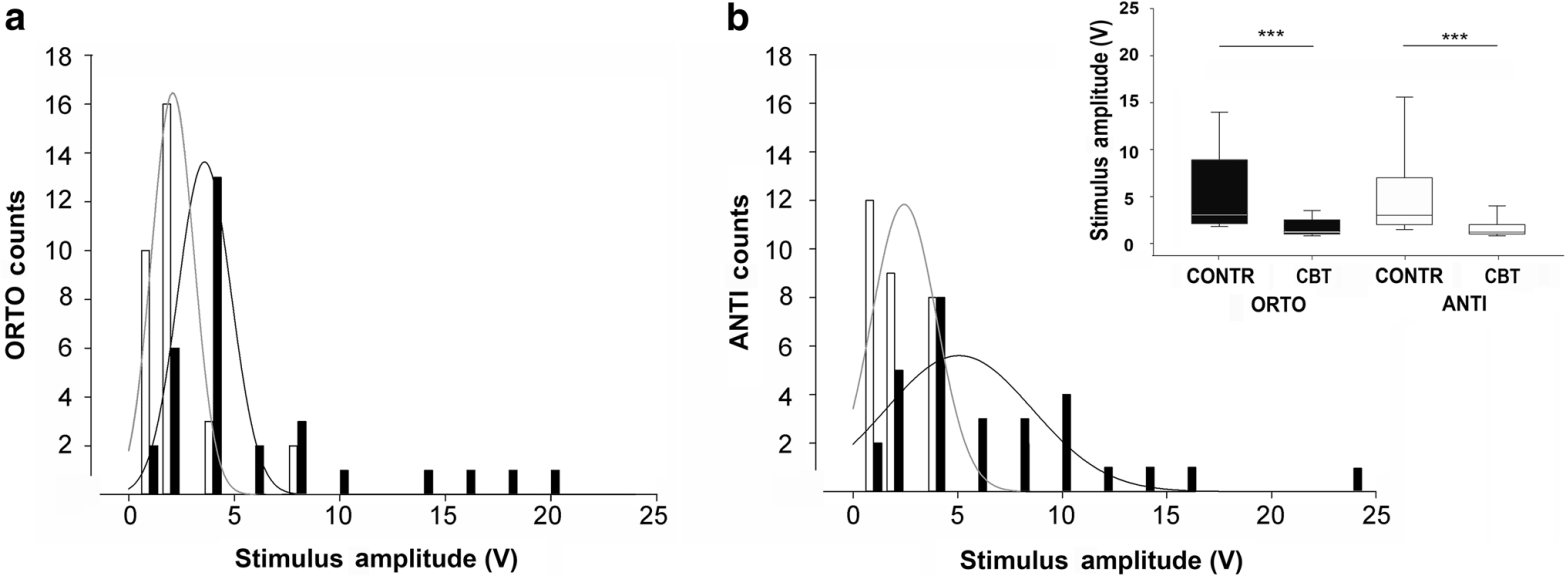
Comparison of the frequency distribution of responses to ORTH and ANTI stimulation. The numbers (counts) of contractions (black) and CBT responses (white) induced by ORTH (**a**) and ANTI (**b**) stimulation is reported for intensities up to 25 V (bin width 0.5V. ORTH n = 31, ANTI *n* = 29). Data were fitted with log-normal three-parameter equations and convergence was satisfied. Inset: box plot of thresholds for ORTH and ANTI contractions (CONTR) and CBT activity (CBT); significant differences were found in both ORTH and ANTI configurations (Mann–Whitney rank sum test, ORTH: ***p < 0.001, *n* = 31; ANTI: ***p < 0.001, *n* = 29)

A frequency distribution analysis was carried out by estimating the number of CBT activity and contraction recorded up to 25 V (bin width of 0.5 V). This showed that contractions were induced at higher stimulation amplitudes than CBT activity in both configurations and for the entire range of stimulus intensities employed (Fig. 3a, b, data fitted with log-normal three-parameter equations; ORTH *n* = 31, ANTI *n* = 29). A cumulative threshold analysis was carried out with data from all the CBT activity and contractions combined in each configuration. There was a statistically significant difference between CBT activity and contraction thresholds in both configurations (Fig. 3 inset, Mann–Whitney rank sum test, ORTH: ***p < 0.001, *n* = 31; ANTI: ***p < 0.001, *n* = 29), supporting the premise that muscle contraction requires recruiting a group of several axons irrespective of the stimulation direction.

### Contraction versus cerebrobrachial tract activity threshold

**Fig. 4:**
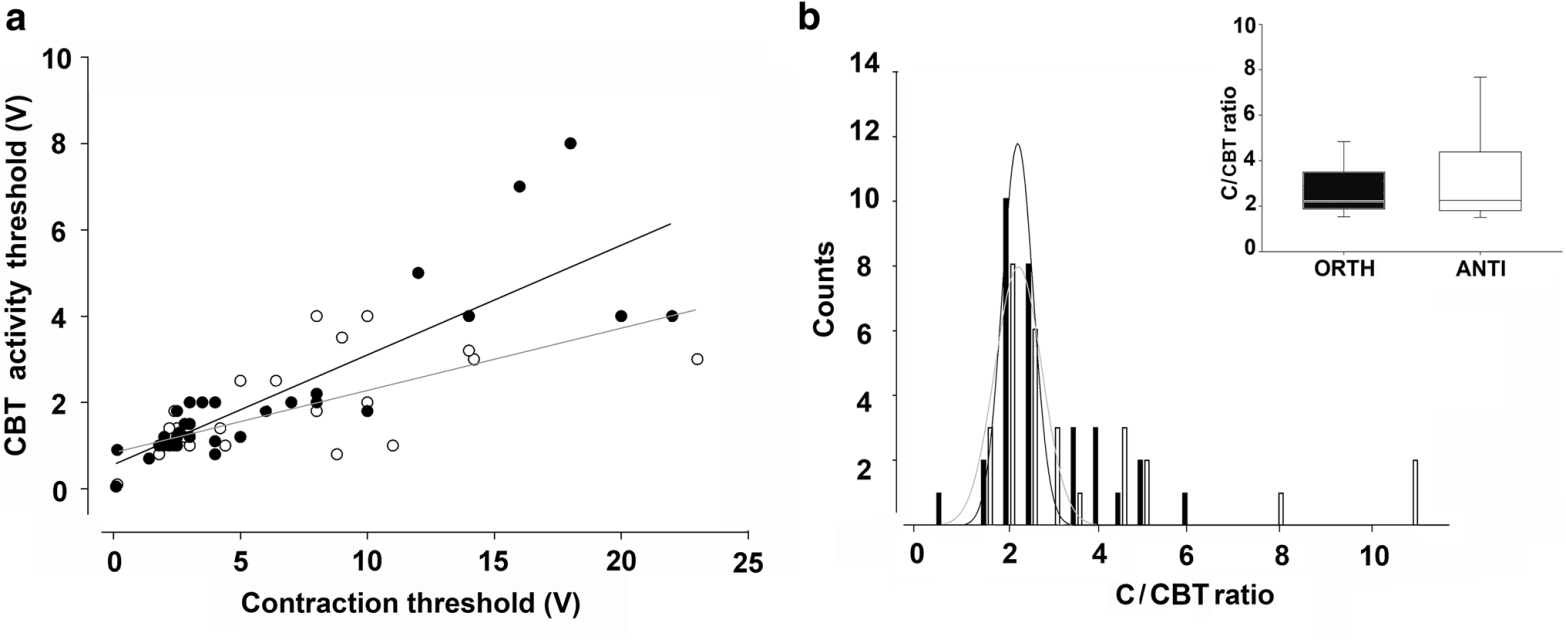
Correlation between CBT activity and contraction threshold. **a** Comparison of CBT activity and contraction threshold pairs showed their linear correlation in both ORTH (filled circles) and ANTI (open circles) (Pearson correlation, ORTH: ρ = 0.845, *p < 0.05, *n* = 31; ANTI: ρ = 0.676, *p < 0.05, *n* = 29). **b** The numbers (counts) of C/CBT ratio induced in ORTH (black) and ANTI (white) are reported for each 0.5 interval of C/CBT ratio. Data were fitted with log-normal three-parameter equations and convergence was satisfied. Inset: box plot of C/CBT ratios in ORTH and ANTI; no significant difference was found between the two configurations (Mann–Whitney rank sum test, *p* > 0.05, *n* = 60)

To uncover possible differences in anterograde and retrograde muscle innervation, we pair compared the CBT activity and contraction thresholds recorded at various locations along the CBT. Trend analysis showed that these datasets were linearly correlated in both ORTH and ANTI (Fig. 4a; Pearson correlation, ORTH: ρ = 0.870, *p* < 0.05, *n* = 31; ANTI: ρ = 0.676, *p* < 0.05, *n* = 29). This linear relationship could be, at least partly, attributed to variability in stimulation effectiveness.

We next explored the relationship between contraction and CBT activity thresholds, expressed as contraction/CBT thresholds (C/CBT) ratio. This ratio normalizes for the effectiveness of stimulation as it indicates the number of axons needed to induce muscle contraction (C) relative to those involved in CBT activity. There was no significant difference in the overall distribution (Fig. 4b, data fitting with log-normal three-parameter equations) and median values of the C/CBT ratio in the two configurations (Fig. 4 inset, Mann–Whitney rank sum test, *p* > 0.05; ORTH *n* = 31, ANTI *n* = 29).

### The spatial distribution of motor axons along the cerebrobrachial tract

**Fig. 5:**
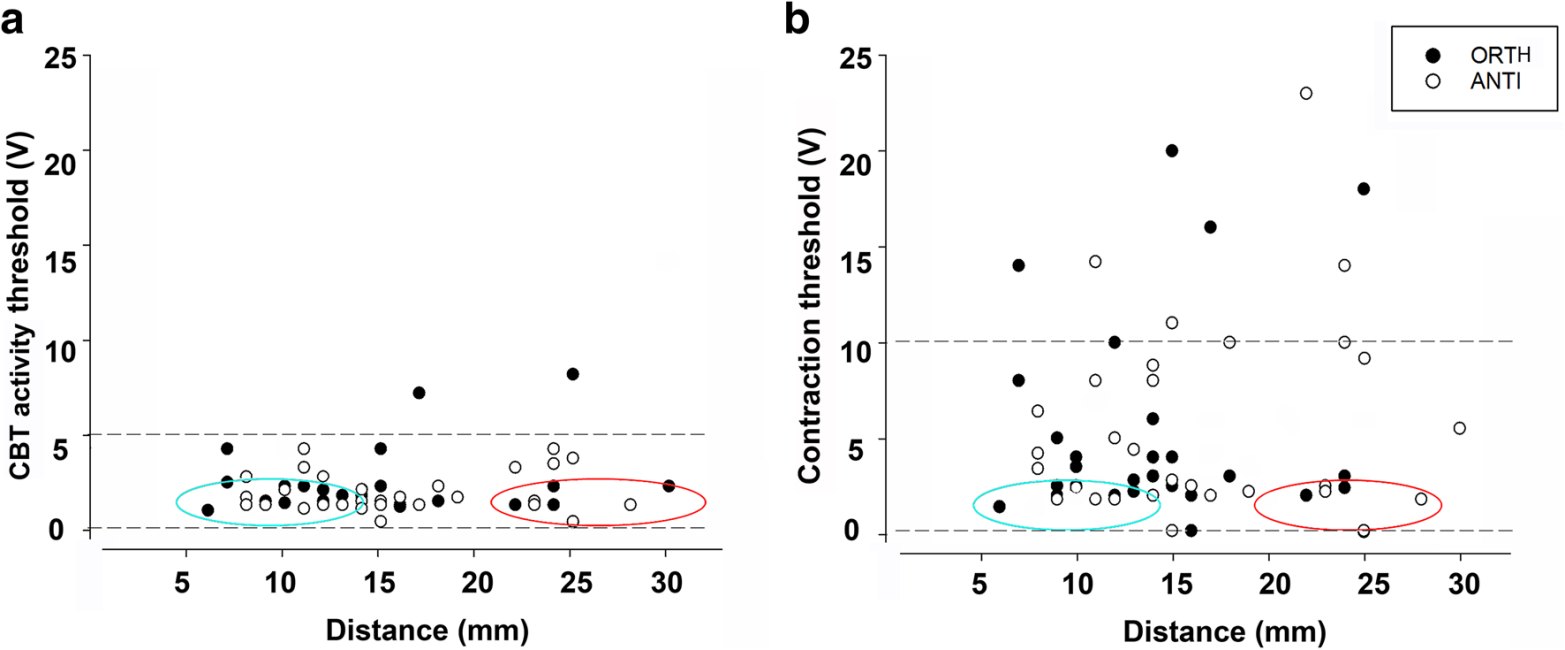
Distribution of high- and low-threshold axons along the ANC. Stimulus thresholds for CBT activity (**a**) and contraction (**b**) are plotted against the distance between the electrodes. Pearson analysis showed no correlation between distance and CBT activity thresholds (Pearson correlation, ORTH: *p* > 0.05, *n* = 31; ANTI: *p* > 0.05, *n* = 29) nor between distance and contraction thresholds (Pearson correlation, ORTH: *p* > 0.05, *n* = 31; ANTI: *p* > 0.05, *n* = 29). Dashed lines mark the voltage range between 0.2 and 5 V (where ~ 90% of the CBT activity was recorded) and between 0.2 and 10V (where ~ 70% of the contractions were obtained). Blue and red ellipses mark low threshold values (~ 0.2 to 2 V) registered close to and far from the recording position, respectively

To test whether axons control specific areas of the arm and to determine their organization along the CBT, we correlated the CBT activity and contraction thresholds with the distance between the stimulating and recording electrodes over a range of 30 mm. CBT activity thresholds were independent of the distance between electrodes in both ORTH and ANTI (Fig. 5a; Pearson correlation, ORTH: *p* > 0.05, *n* = 31; ANTI: *p* > 0.05, *n* = 29). Most recordings (~ 90%) were obtained at stimulation intensities between 0.2 and 5 volts (Fig. 5a, dashed lines). Low-threshold CBT activity (~ 0.2 to 2 V) was induced at both closer and farther locations from the recording position (cf. points within red and blue ellipses in Fig. 5a). This indicates that a relatively large proportion of low-threshold axons may run for rather long distances along the CBT. This transmission line organization seems to be similar in both anterograde and retrograde directions (Fig. 5a filled and open circles, respectively).

To study the spatial pattern of motor innervation along the arm, we correlated contraction thresholds with the distance between the electrodes. We found no correlation between them (Fig. 5b; Pearson correlation, ORTH: *p* > 0.05, *n* = 31; ANTI: *p* > 0.05, *n* = 29). Most contractions (~ 70%) were obtained at stimulus values between 0.2 and 10 volts (Fig. 5b dashed lines) and low-threshold contractions (~ 0.2 to 2 V) were evoked at both closer and farther locations from the recording electrode in both ORTH and ANTI configurations (cf. points within red and blue ellipses in Fig. 5b). This suggests that a substantial proportion of motor axons may run for rather long distances along the CBT. The fact that there was no clear difference between the ANTI and ORTH stimulation (Fig. 5b) suggests that these low-threshold motor axons in the CBT innervate motor neurons *en passant* as they pass through the MC ganglia.

### Velocity of signal transmission

**Fig. 6:**
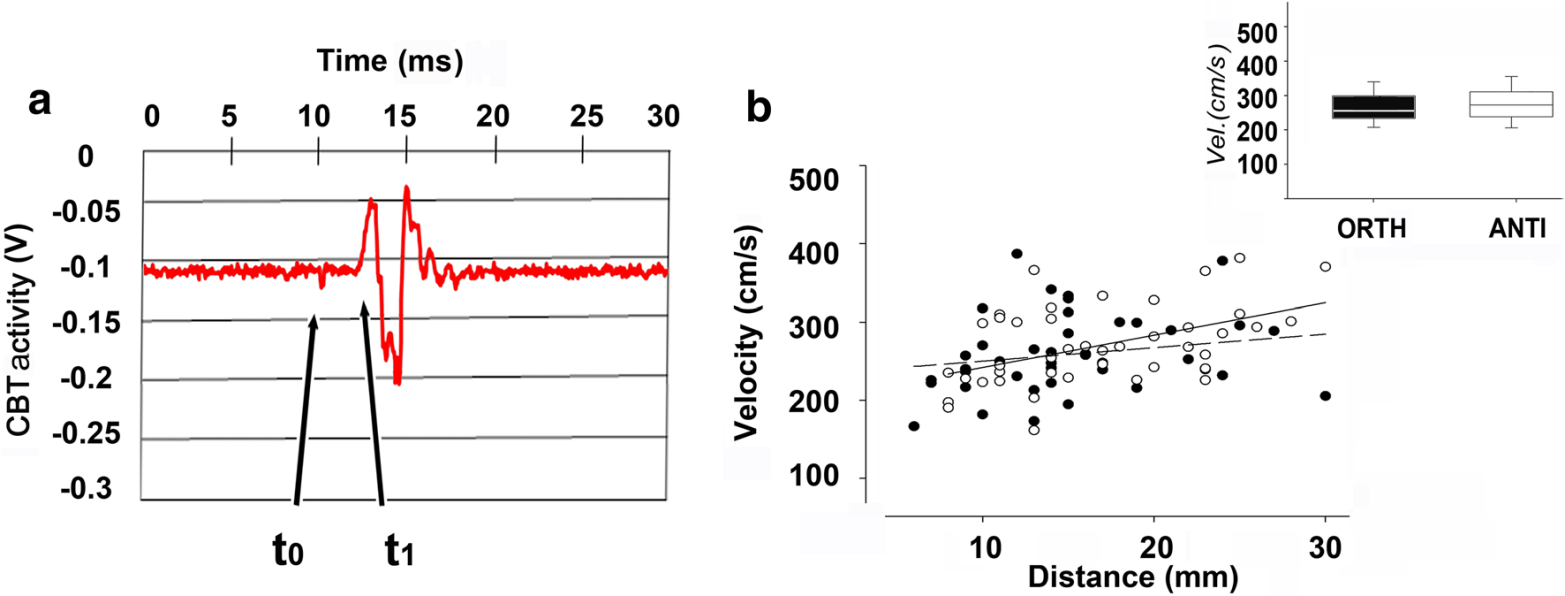
Velocity of transmission of CBT activity. **a** Signal propagation velocity was calculated from the delay between the stimulus artifact at *t*_0_ and the first positive peak at t_1_ of the compound recording. **b** Signals propagated at a constant velocity of ~ 250–300 cm/s along the ANC in both ORTH (filled circles, *n* = 46) and ANTI (open circles, *n* = 45) direction. Inset: box plot of velocities in ORTH (black) and ANTI (white); no significant difference was found between the two configurations (*t* test, *p* > 0.05, *n* = 91)

Using the delay between stimulation onset and the first positive peak of the compound spike recorded in the CBT (Fig. 6a), we estimated the velocity of signal transmission to be ~ 250–300 cm/s (Fig. 6b). This range is compatible with that of unmyelinated axons with relatively large diameters (Bullock and Horridge 1965). The average, variance, maximum and minimum velocities were independent of the distance between the electrodes and of the conduction orientation (see inset Fig. 6b, *t* test, *p* > 0.05, *n* = 91). These data support the existence of long uninterrupted transmission lines along the CBT in both directions along the arm.

### Morphological pattern of cerebrobrachial tract innervation

Confocal investigation of NF200 immunostained arm sagittal sections revealed an interesting pattern of innervation in which CBT fibers leave their main bundle along the axonal tract to enter a ganglionic structure (Online resource 1 a) and where a single bundle of axons can innervate at the same time a different portion of the medullary cord such as ganglionic and inter-ganglionic areas (Online resource 1 b–e). Yet, the resolution and complexity of innervation do not allow showing clear morphological characteristics of *en passant* or of direct pattern of innervation. The existence of this complex organization has been previously assessed by Graziadei although he did not attempt to describe the specific pattern of innervation of single ganglia (Graziadei 1971).

## Discussion

Octopuses can initiate stereotypical movements practically at any point along the arm (Gutfreund et al. 1996; Sumbre et al. 2001, 2005, 2006). Earlier work on the organization of the arm neural circuitry (Rowell 1963, 1966; Young 1971; Budelmann and Young 1985) and more recent studies (Sumbre et al. 2001; Gutfreund et al. 2006) have led to the suggestion that the motor command axons in the cerebrobrachial tracts (CBT) are organized as “labeled lines” innervating specific locations along the arm neuromuscular system (schematically summarized in Fig. 1). The aim of this work was to test this hypothesis.

The arm neural network is composed of two dorsal (aboral) CBT that transmit information locally, and to and from the higher centers. The CBT axons do not directly innervate the arm muscles; rather they innervate motoneuronal circuits located in the ganglion-like medullary cord (MC) ventral (oral) to the CBT (Fig. 1). The MC receives sensory inputs from the suckers and skin, and proprioceptive information from the muscles and it sends motor outputs via specific lateral roots to the intrinsic arm muscles (Cate 1928; Graziadei 1971; Matzner et al. 2000; Gutfreund et al. 2006). With this organization, a labeled line addressing a certain MC location (schematized by green arrows in Fig. 1) may activate a confined motor neuron pool to create a bend or stiffness at a designated location along the arm. Note that in this case, only orthodromic (ORTH) stimulation should initiate local contraction. The involvement of labeled lines is not supported by our findings because stimulation in both anterograde and retrograde directions initiated muscle contraction. Yet, we cannot rule out the possibility that a relatively small fraction of the axons in the CBT is organized as labeled lines as, for example, in the chromatophore system (Messenger 2001; Liu and Chiao 2017).

As the minimum threshold level varied independent of the distance between the electrodes and the direction of stimulation, there appear to be fast axons running for at least 30 mm along the CBT (the distance tested here). That is, the CBT appears to contain a large group of low-threshold axons that functionally innervate long sections of the arm MC *en passant* and can, theretofore, activate the motor neurons of the intrinsic arm muscles independent of stimulus orientation (red arrows, Fig. 1). This interpretation fits with Gutfreund et al. (2006) showing that the activity in the lateral motor roots is locked to the activity of the fast propagating axonal tracts of the CBT (Gutfreund et al. 2006). The advantage of the *en passant* innervation may be seen in actions such as stiffening. This requires controlling the simultaneous contraction of a large part of the arm musculature to create a dynamical skeletal structure essential for generating movements in muscular hydrostats. Arm stiffening is important in bend propagation (Gutfreund et al. 1998; Sumbre et al. 2001; Yekutieli et al. 2005a, b), walking (Huffard et al. 2005; Huffard 2006; Levy and Hochner 2017) and in creating pseudo-articulated structures during fetching (Sumbre et al. 2005, 2006; Yekutieli et al. 2002), all widely used movements in the octopus’s behavioral repertoire.

How can the octopus “select” a specific arm section to use within a certain task without using labeled lines? One possibility is that task-specific requirements result in local sensory information being integrated with a specific central command to generate the arm behavior at the site “labeled” by the peripheral sensory input. For example, in fetching food to the mouth, the position of the sensory stimulus determines the location for forming joints that shape the arm into an ad hoc quasi-articulated arm structure, allowing the arm to fetch accurately (Sumbre et al. 2005, 2006). The integration of central *en passant* commands with local signals also fits the control of other arm movements and autonomous reflexes (Cate 1928; Altman 1971), where the central brain sends efferent signals to activate peripheral motor programs embedded in the elaborate neuromuscular system of the arm itself (Sumbre et al. 2001; Gutfreund et al. 2006; Zullo et al. 2009; Zullo and Hochner 2011). The integration between sensory and motor information may take place in the MC where the motor circuits lie (Graziadei 1971; Gutfreund et al. 2006). This would be in agreement with JZ Young’s intelligent inference based on the relatively small number of efferents/afferents running between the CNS and the periphery.

We, therefore, propose this *en passant* distribution of motor commands as a novel motor control mechanism that may fit the motor control of the long and flexible *Octopus vulgaris* arms, whose central representation is not organized as in vertebrates in body-part coordinates (Zullo et al. 2009). Our findings further support the idea that motor control of this soft-bodied animal involves a unique embodied organization of the interplay between the nervous system, the body morphology, and the animal’s task environment (Zullo and Hochner 2011; Hochner 2012, 2013; Levy and Hochner 2017).

## Electronic supplementary material

Below is the link to the electronic supplementary material.

**Online resource 1**. CBT morphological pattern of innervation. **a** Mosaic reconstruction of confocal images of an arm sagittal section stained with NF200 and revealing the complex organization of CBT axonal bundles (green arrows indicate bifurcation points along the bundle). Blue and yellow rectangles enclose interganglionic and ganglionic areas respectively (scale bar 50 μm). **b-e** Magnifications of two Z stacks of the blue (**b-c**) and yellow (**d-e**) areas in a showing how fibers from the same nerve bundle can reach various part of the underlying MC such as interganglionic (see white arrows in **b-c**) and the ganglionic areas (see white arrows in **d-e**) (scale bar 10 μm). (TIF 13662 KB)

## Abbreviations

ANC: Axial nerve cord
ANTI: Antidromic
CBT: Cerebrobrachial tracts
C/CBT: Contraction/CBT thresholds
L: Longitudinal
MC: Medullary cord
C: Muscle contraction
NF: Neurofilaments
O: Oblique muscles
ORTH: Orthodromic
RT: Room temperature
TR: Trabeculae
T: Transverse muscles
