# Supplementary figures and images for: Motor control pathways in the nervous system of Octopus vulgaris arm
Source: J Comp Physiol A Neuroethol Sens Neural Behav Physiol. 2019 Mar 27;205(2):271–9. doi: 10.1007/s00359-019-01332-6 (PMC6478645; doi:10.1007/s00359-019-01332-6)

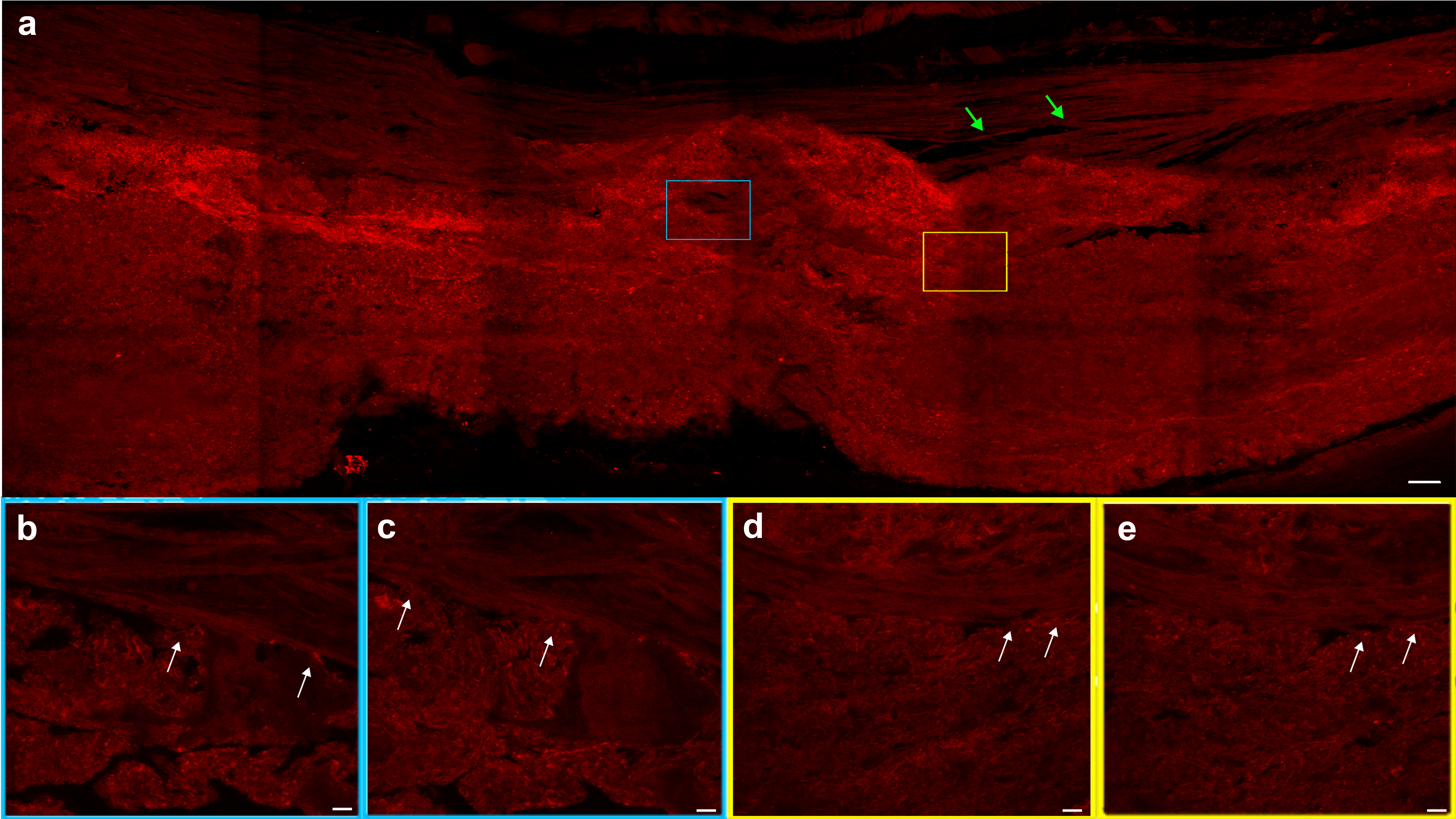

Supplement: Supplementary file 1 — Online resource 1. CBT morphological pattern of innervation. a Mosaic reconstruction of confocal images of an arm sagittal section stained with NF200 and revealing the complex organization of CBT axonal bundles (green arrows indicate bifurcation points along the bundle). Blue and yellow rectangles enclose interganglionic and ganglionic areas respectively (scale bar 50 μm). b-e Magnifications of two Z stacks of the blue (b-c) and yellow (d-e) areas in a showing how fibers from the same nerve bundle can reach various part of the underlying MC such as interganglionic (see white arrows in b-c) and the ganglionic areas (see white arrows in d-e) (scale bar 10 μm). (TIF 13662 KB) [file 359_2019_1332_MOESM1_ESM.tif]
